# Genetic Polymorphisms of* IL1B, IL6,* and* TNFα* in a Chinese Han Population with Pulmonary Tuberculosis

**DOI:** 10.1155/2018/3010898

**Published:** 2018-05-14

**Authors:** Shouquan Wu, Yu Wang, Miaomiao Zhang, Saurav S. Shrestha, Minggui Wang, Jian-Qing He

**Affiliations:** ^1^Department of Respiratory and Critical Care Medicine, West China Hospital, Sichuan University, Chengdu, Sichuan, China; ^2^Department of Radiology, West China Hospital, Sichuan University, Chengdu, Sichuan, China

## Abstract

**Background:**

The factors that predispose to pulmonary tuberculosis (PTB) are not fully understood. Previous studies have shown that cytokine gene polymorphisms were associated with PTB.

**Objectives:**

In this study, we have investigated the relationship between* ILB*,* IL6,* and* TNFα* polymorphisms and a predisposition to* Mycobacterium tuberculosis* (MTB) infection and PTB.

**Methods:**

A total of 209 cases of PTB, 201 subjects with latent TB infection (LTBI), and 204 healthy controls (HCS) were included in this study. Logistic regression analyses under allelic, homozygous, and heterozygous models were used to calculate *P* values, odds ratios (ORs), and 95% confidence intervals (CIs) for assessing the association between single nucleotide polymorphisms (SNPs) and disease risk, adjusting for sex and age. Genotyping was conducted using the improved multiplex ligase detection reaction (iMLDR) method.

**Results:**

When comparing PTB patients with LTBI subjects, significant associations with disease development were observed for SNPs of* IL6* and* TNFα*. When comparing LTBI subjects with HCS,* IL1B* polymorphisms were significantly associated with LIBI. Haplotype analyses suggested that the CGG haplotype of* IL1B* was associated with an increased risk of PTB (*P* = 0.039, OR = 1.34, 95% CI: 1.01–1.76), while the TTGCG haplotype of* TNFα* was a protective factor against PTB (*P* = 0.039, OR = 0.66, 95% CI: 0.44–0.98).

**Conclusion:**

Our study demonstrated that* IL1B* variants were related to LTBI and* IL6* and* TNFα* variants were associated with PTB.

## 1. Introduction

Tuberculosis (TB) is still a major cause of mortality and morbidity in developing countries. In 2015, there were an estimated 9.2 million new TB cases [1]. India, Indonesia, and China had the largest number of patients with TB: 23%, 10%, and 10% of the global total, respectively. It is reported that one-third of the world's population is infected with* Mycobacterium tuberculosis* (*MTB*); however, only 10% of the* MTB* infected population will develop TB disease during their lifetime. TB is mainly caused by* MTB* that primarily invades the lung phagocytes such as macrophages, neutrophils, monocytes, and dendritic cells [2]. In 90% of subjects infected with* MTB* (LTBI), the ensuing immune response arrests further the development of TB disease. However, the predisposing factors for TB remain unclear. Previous studies have demonstrated that various host genetic polymorphisms play important roles in determining TB susceptibility [3, 4].

Investigating the various host genes involved in susceptibility and resistance to TB may enhance understanding of the pathogenesis of TB and development of treatment or prophylactic strategies.

Previous studies have increased our understanding of the roles of host genetics in susceptibility to* MTB* infection [4]. There are several cytokines and their receptors expressed by macrophages that are important for host responses to TB. They include tumor necrosis factor (TNF), interleukin-1*β* (IL1B), and IL6 [5]. When* MTB* invades the lungs, these cytokines are released at the site of disease after the interaction between T lymphocytes and infected macrophages. Macrophages are essential for the induction of the immune response against* MTB*. When* MTB* enters the lungs, macrophages are among the first to sense it. After the phagocytosis of* MTB* by lung macrophages, the process generates an inflammatory response and antigen presentation to effector T cells [6]. Furthermore, macrophages can kill phagocytosed* MTB* and are involved in tissue repair as a consequence of the inflammatory process [7].

Several cytokine gene polymorphisms have been reported to affect gene transcription and influence serum cytokine production [8, 9]. Polymorphisms in cytokine gene have been suggested to play key roles in the susceptibility, development, and severity of infectious diseases [10]. Among the inflammatory cytokine genes,* IL1B*,* IL6*, and* TNFα* were demonstrated to be associated with TB infection or TB disease [11–13].

We therefore investigate the impact of the most widely studied SNPs of the three genes on LTBI and/or PTB. Participants in our study formed three groups: healthy control subjects without* MTB* infection (HCS), latent TB infection (LTBI), and bacteria-confirmed active pulmonary TB disease (PTB).

## 2. Methods

### 2.1. Study Population

A total of 209 patients with PTB and 415 close contacts of people with active PTB were enrolled between 2013 and 2014 in the West China Hospital of Sichuan University, Chengdu, China. These subjects were all Chinese Han individuals. Close contacts of active PTB cases included spouses, accompanying people, and coworkers. Some medical staff of the TB ward was also included. After one year of follow-up, several close contacts developing into PTB were excluded. We divided the remaining 405 close contacts into LTBI and HCS according to interferon gamma release assay (IGRA) results at enrolment, chest X-ray, and symptoms at enrolment and at one-year follow-up.

The definition of close contacts is as follows: (1) shared airspace with PTB for at least 15 hours per week for at least one week during an infectious period and (2) shared airspace with a PTB patient for at least 180 hours during an infectious period. The infectious period was 3 months before the collection of the first culture-positive sputum or the date of first signs of cough (whichever was earlier) until two weeks after the TB combination treatment therapy. LTBI controls had no TB-related symptoms but a positive IGRA test. HCS without* MTB* infection were IGRA negative and without evidence of active TB and history of TB. PTB patients were diagnosed by sputum positive and/or through bronchoscopic biopsy and pathological examinations. IGRA was performed according to the manufacturer's instructions (Beijing Wantai DRD Co., Ltd., Beijing, China). All participants in our study were older than 18 years and younger than 80 years. All participants with blood relationship were excluded from this genetic association study. Subjects with autoimmune diseases, chronic obstructive pulmonary disease, diabetes, hepatitis, and those tested positive for HIV were excluded from the study. All participants were required to provide their written informed consent to participate in this study. Specialized nurses drew 2–5 mL of blood with anticoagulant from all participants and then the blood was preserved in an 80°C freezer for DNA extraction and genotyping.

### 2.2. SNP Selection and Genotyping

SNPs were selected based on a literature review of previous studies and in silico functional prediction from the NIH FuncPred website (https://snpinfo.niehs.nih.gov/snpinfo/snpfunc.html). Only those SNPs with potential effects on function and significant disease associations were selected in our study. As a result, there were 11 SNPs from 3 target genes that were selected for this study. Among the 11 SNPs, rs1143623 [14], rs2069837 [15], rs1799724 [16], rs1800629 [16], and rs1800630 [16] were associated with transcriptional activity. rs1143634 [17], rs16944 [18], and rs1800795 [19] polymorphisms were found to regulate cytokine levels. rs1799964 [20] and rs361525 [21] were found to be contributory factors to* TNFα *gene expression; rs17147230 was predicted to have functional significance by the FuncPred algorithm.

Genomic DNA was isolated using a genomic DNA purification kit (Axygen Scientific Inc., Union City, CA, USA) according to the manufacturer's instructions. Genotyping was conducted using the improved multiplex ligase detection reaction (iMLDR), with technical support from the Shanghai Genesky Biotechnology Company. To validate the genotype results, 5% of the samples were repeated by iMLDR.

### 2.3. Statistical Analysis

Hardy-Weinberg equilibrium (HWE) was estimated using the *x*^2^ test. Differences in age between cases and controls were compared by an unpaired *t*-test. Differences in the distribution of sex between the two groups were assessed using the *x*^2^ test. Multiple logistic regression under allelic (minor allele versus major allele), homozygous (homozygous minor genotype versus homozygous major genotype), and heterozygous (heterozygous genotype versus homozygous major genotype) models was used to calculate *P* values, odds ratios (ORs), and 95% confidence intervals (CIs) for assessing the association between SNPs and disease risk, adjusting for sex and age. Haplotype association was calculated by SHEsis online software (http://analysis.bio-x.cn/). *P* values < 0.05 were considered statistically significant. Statistical analyses were performed using SPSS (SPSS Inc., Chicago, IL, USA) 17.0 software.

## 3. Results

### 3.1. Demographic and Clinical Characteristics of Study Subjects and Results of Quality Control

The study subjects consisted of 209 PTB patients, 201 LTBI, and 204 HCS (Table 1). The distributions of sex in two groups had no significant difference. However, the mean ages of the three groups were significantly different (Table 1).

The characteristics of all the SNPs were showed in Table 2. One SNP (rs1800795 of* IL6*) was out of HWE in the HCS group and was excluded from further analysis. The remaining 10 SNPs did not deviate from HWE in the control subjects (*P* > 0.05).

### 3.2. Association between 10 SNPs in the* IL1B, IL6*, and* TNFα* Genes and LTBI or TB Susceptibility

Ten SNPs in the* IL1B*,* IL6*, and* TNFα* genes were evaluated for associations with LTBI or PTB. The genotype distributions in LTBI were compared with HCS and PTB cases, respectively. Differences in genotype distribution between the study groups are shown in Table 3. Significant associations were found for* IL1B* rs1143634 between LTBI and HCS. Compared with the* IL1B* rs1143634G allele, allele A had an 82% decreased risk for development LTBI (*P* = 0.029, OR = 0.18, 95% CI: 0.039–0.84). The rs1143634GA genotype was also a protective factor for LTBI (*P* = 0.029, OR = 0.18, 95% CI: 0.08–0.83). We found the rs2069837 polymorphism of* IL6* was associated with PTB. Compared with AA, GA had a significantly decreased risk of LTBI developing into PTB (*P* = 0.04, OR = 0.62, 95% CI: 0.4–0.98). A significantly decreased risk of PTB development was also observed for the* TNFα* rs1799724T allele compared with C allele (*P* = 0.039, OR = 0.64, 95% CI: 0.42–0.98).

### 3.3. Association between Haplotypes of* IL1B*,* IL6, TNFα,* and LTBI or TB Susceptibility

Haplotype association analyses suggested that the rs1143623-rs1143634-rs16944 CGG haplotype of* IL1B* was a risk factor for PTB development (*P* = 0.039, OR = 1.34, 95% CI: 1.01–1.76), while the rs1799724-rs1799964-rs1800629-rs1800630-rs361525 TTGCG haplotype of* TNFα *was a protective factor against PTB development (*P* = 0.039, OR = 0.66, 95% CI: 0.44–0.98) (Table 4).

## 4. Discussion

Most previous studies focused on the association between gene polymorphisms and TB using control subjects including LTBI and no TB infection individuals. However, few studies have identified candidate genes related to LTBI and/or TB. In this study, we designed three study groups including HCS without TB infection, LTBI, and PTB to identify risk factors for both LTBI and TB and hence find genetic markers specific for TB development stages. When comparing PTB with LTBI controls, significant relationships were detected for* IL6* rs2069837 G/A and* TNFα *rs1799724 C/T. When comparing LTBI with HCS, significant relationships were detected for* IL1B *rs1143634GA.

It is well recognized that proinflammatory cytokines, including IL1B, IL6, and TNF*α*, have been implicated in the host defense against* MTB* infection. IL1B, produced by macrophages and monocytes, is involved in the immune response and is an important proinflammatory cytokine [22]. IL1B plays a critical role in the early stages of antimycobacterial immune response and protects the tissue by activating epithelial antimicrobial peptides [23]. IL1B not only regulates some immune cells but also stimulates expression of other inflammation-associated proteins [24]. Previous studies have revealed that high levels of IL1B were detected in alveolar macrophages from PTB patients and these levels were related to significant tissue necrosis in lung lesions of PTB cases with a large cavity [25]. Thus, polymorphisms of* IL1B* have become biologically probable candidate markers associated with PTB susceptibility. Association studies of* IL1B *polymorphisms and TB have previously been conducted. Hall et al. researched the relationship between 29 candidate genes related to innate immune responses and suggested that rs1143643 and rs1143633 in* IL1B *were associated with TB [13]. A meta-analysis including 3327 participants indicated that rs16944 and rs1143634 alleles/genotypes in* IL1B *have no relationship with TB [26]. However, these studies only researched the genetic polymorphisms and the development of TB utilizing controls without differentiating HCS from LTBI. In the present study, we identified rs1143634 allele/genotype was associated with LTBI development compared with HCS but not with LTBI. This inconsistent result may be attributed to the different type of control group. A previous study suggested that the use of different controls could result in contrary conclusions and it is important to divide the controls into LTBI and HCS to understand the role of these genes in TB pathogenesis and progression [4].

IL6 is a pleiotropic proinflammatory cytokine, and high levels of IL6 were observed in TB patients [27]. To show the importance of IL6 in TB, murine models with mycobacterial infections and models with a TB subunit vaccine indicated that IL6 was essential for the development of an optimal T-cell response [28]. IL6 mRNA and protein expression were significantly elevated in the serum of PTB patients [29].* MTB* stimulates the secretion of IL6 which inhibits IFN-signaling, thus enabling* MTB* to evade eradication by a cellular immune response [30]. Previous studies have investigated the relationship between* IL6 *polymorphisms and TB; however, the results were inconsistent [30, 31]. A meta-analysis indicated that* IL6* rs1800795 was associated with decreased risk of TB [32]. Unfortunately* IL6* rs1800795 diverged from the HWE in our data and as a result was excluded from our study. Our study revealed that rs2069837GA was associated with decreased risk of TB. Moreover, our study revealed that this polymorphism was associated with PTB when compared with LTBI. SNP rs2069837 is located within the second intron of* IL6* and was not in linkage disequilibrium (LD) with any other SNPs in our study. LD was also not found in the LD data from the HapMap project (http://hapmap.ncbi.nlm.nih.gov/). This explains why only a single variant of* IL6* was found as a protective factor for PTB. rs2069837 is located within a regulatory genetic region [33], indicating this genetic variant may modify risk of PTB. The rs2069837 polymorphism has been suggested to be related to inflammatory diseases such as Takayasu arteritis [33]. Consistent with this observation, our results indicated that this genetic variant was related to PTB which is also an inflammatory disease. However, we demonstrated that this SNP was a protective factor for PTB. This phenomenon may be due to the different MAF between the two ethnic groups.

When infected with* MTB*, a strong inflammatory cell-mediated immune response appears, with elevated expression* TNFα *[34]. TNF*α* was not only an important component of the immune response to TB but was also involved in granuloma formation [35]. There have been a number of studies of the association of* TNFα *polymorphisms and TB, however; the results were inconsistent [36]. It was reported that the* TNFα *rs1800629 and rs361525 polymorphisms were not related to TB [36, 37]. These two genetic variants were also found to have no relationship with PTB/HCS in our study. Our study demonstrated that the* TNFα *rs1799724 allele T was associated with decreased risk of PTB. Since* TNFα *rs1799724T had higher transcriptional activity and an increase in TNF*α* cytokine production [16], we speculated this genetic variant was associated with* TNFα *gene expression. Lindenau et al. showed that the* TNFα *rs1799724 polymorphism had no significant difference in allele/genotype distribution between TB patients and non-TB controls. However, they observed an association between tuberculin skin test (TST) reactivity and haplotypes derived from* TNFα *rs1799724 [38]. Further studies are warranted to verify our results.

Previous studies have mainly focused on the association between gene polymorphisms and TB. However, most of these investigations employed healthy controls without identifying* MTB* infection status. Recent research has found that some genes may be associated with LTBI but not active TB [36, 39], and our results have also shown that the* IL1B* polymorphism was only associated with LTBI. In addition, other studies have reported that some genes may differentiate between LTBI and active TB disease [40, 41]. The aforementioned results are important in order to further understand the important role of SNPs in TB progression. Such information may also help in the differentiation between LTBI and PTB and hence affect therapeutic regimen. We reasoned that the genetic susceptibility to TB is important not only for the general population but also for the patients with autoimmune diseases under immunosuppressive treatments such as treatment with anti-*TNFα* or anti-IL6 antibodies. However, most researchers excluded participants with autoimmune diseases from their study [36], and thus it is difficult to directly evaluate the role of genetic variants in the diagnosis of LTBI for patients under immunosuppressive treatment. Therefore, more studies regarding the association of SNPs with LTBI in patients treated with such biological therapies are needed to verify our results.

Nevertheless, our research has several limitations. First, functional validation of the associated SNPs was not conducted. As a result, the true causal allele underlying the genetic association result is still unknown. Second, the results have not been validated in a replication cohort, which may increase the chance of type I errors. And finally, we did not analyze the association between SNPs and detailed clinical characteristics.

In conclusion, our study demonstrated that* IL6* and* TNFα *genetic polymorphisms were significantly associated with susceptibility to PTB in LTBI subjects. We also identified that* IL1B *polymorphisms were associated with LTBI. Our results may reflect the genetic aspects of a predisposition to different stages of developing PTB and these results warrant verification by larger studies.

## Figures and Tables

**Table 1:** Baseline information on the study groups.

	PTB (*n* = 209)	LTBI (*n* = 201)	HCS (*n* = 204)	PTB versus LTBI *P* value	LTBI versus HCS *P* value
Age (mean ± SD)	38.76 ± 16.97	49.09 ± 15.91	45.71 ± 14.90	<0.001	0.027
Sex (male) *N* (%)	107 (0.51)	95 (0.47)	93 (0.46)	0.431	0.765

PTB, pulmonary tuberculosis; LTBI, latent tuberculosis infection; HCS, healthy controls.

**Table 2:** Characteristics of the SNPs in our study.

Gene/SNPs	chromosome	Location on chromosome	Location in the gene	MA	MAF	MA	MAF	HWE	
				LTBI		HCS		LTBI	HCS
*IL1B*									
rs1143634	2	113590390	Nonsynon_exon 5	A	0	A	0	0.99	0.94
rs16944	2	113594867	5′FLANKING	A	0.44	A	0.51	0.44	0.19
rs1143623	2	113595829	5′FLANKING	G	0.57	G	0.6	0.97	0.97
*IL6*									
rs17147230	7	22762176	Intergenic	T	0.46	T	0.49	0.88	0.58
rs1800795	7	22766645	5′FLANKING	C	0.01	C	0.01	0.99	<0.01^*∗*^
rs2069837	7	22768027	intron2	G	0.19	G	0.22	0.2	0.98
*TNFα*									
rs1799964	6	31542308	5′FLANKING	C	0.18	C	0.16	0.91	0.79
rs1800630	6	31542476	5′FLANKING	A	0.15	A	0.14	0.96	0.99
rs1799724	6	31542482	5′FLANKING	T	0.11	T	0.17	0.76	0.69
rs1800629	6	31543031	5′FLANKING	A	0.06	A	0.08	0.94	0.98
rs361525	6	31543101	5′FLANKING	A	0.03	A	0.03	0.92	0.08

SNP, single nucleotide polymorphism; LTBI, latent tuberculosis infection; HCS, healthy controls; MA, minor allele; MAF, minor allele frequency; HWE, Hardy Weinberg equilibrium. ^*∗*^Due to HWE < 0.05, it was excluded from our study.

**Table 3:** Distributions of cytokine genotypes in the three groups.

Gene: SNPs	PTB *N* = 209 (%)	LTBI *N* = 201 (%)	HCS *N* = 204 (%)	PTB versus LTBI *P*^#^ value	OR (95% CI)^#^	LTBI versus HCS *P*^#^ value	OR (95% CI)^#^
*IL1B*							

rs1143623 C>G							
Genotypes							
CC	75 (35.9)	63 (31.5)	74 (36.3)	Reference			
GC	104 (49.8)	100 (50.0)	96 (47.1)	0.732	0.92 (0.59–1.46)	0.424	1.20 (0.77–1.86)
GG	30 (14.4)	37 (18.5)	34 (16.7)	0.239	0.69 (0.37–1.28)	0.436	1.26 (0.71–2.45)
Alleles							
C	254 (60.8)	226 (56.5)	244 (59.8)	Reference			
G	164 (39.2)	174 (43.5)	164 (40.2)	0.277	0.85 (0.63–1.14)	0.378	1.14 (0.90–1.50)
rs1143634 G>A							
Genotypes							
GG	205 (98.1)	198 (99.0)	194 (95.0)	Reference			
GA	4 (1.9)	2 (1.0)	10 (5.0)	0.398	2.18 (0.36–13.3)	0.028^*∗*^	0.18 (0.08–0.83)
AA	0 (0)	0 (0)	0 (0)	-		-	-
Alleles							
G	414 (99.0)	398 (99.0)	398 (98.0)	Reference			
A	4 (1.0)	2 (1.0)	10 (2.0)	0.400	-	0.029^*∗*^	0.18 (0.039–0.84)
rs16944 G>A							
Genotypes							
GG	61 (29.2)	43 (21.5)	47 (23.0)	Reference			
GA	110 (52.6)	109 (54.5)	115 (56.4)	0.321	0.78 (0.47–1.27)	0.981	0.99 (0.61–1.63)
AA	38 (18.2)	48 (24.0)	42 (20.6)	0.108	0.61 (0.33–1.12)	0.619	1.17 (0.64–2.14)
Alleles							
G	232 (55.5)	195 (48.8)	209 (51.2)	Reference			
A	186 (44.5)	205 (51.3)	199 (48.8)	0.108	0.79 (0.59–1.05)	0.569	1.08 (0.82–1.43)

*IL6*							

rs2069837 A>G							
Genotypes							
AA	144 (68.9)	129 (64.5)	125 (61.3)	Reference			
GA	56 (26.8)	68 (34.0)	70 (34.3)	0.041^*∗*^	0.62 (0.40–0.98)	0.812	0.95 (0.63–1.44)
GG	9 (4.3)	3 (1.5)	9 (4.4)	0.303	2.11 (0.51–8.78)	0.085	0.31 (0.08–1.18)
Alleles							
A	344 (82.3)	326 (81.5)	320 (78.4)	Reference			
G	74 (17.7)	74 (18.5)	88 (21.6)	0.302	0.82 (0.56–1.20)	0.611	0.92 (0.66–1.28)
rs17147230 A>T							
Genotypes							
AA	64 (30.6)	56 (28.0)	49 (23.9)	Reference			
AT	103 (49.3)	103 (51.5)	110 (53.7)	0.580	0.87 (0.54–1.41)	0.438	0.83 (0.52–1.33)
TT	42 (20.1)	41 (20.5)	46 (22.4)	0.448	0.79 (0.43–1.45)	0.435	0.80 (0.45–1.42)
Alleles							
A	231 (55.3)	215 (53.8)	208 (50.7)	Reference			
T	187 (44.7)	185 (46.2)	202 (49.3)	0.494	0.9 (0.68–1.21)	0.666	0.94 (0.72–1.23)

*TNFα*							

rs1799724 C>T							
Genotypes							
CC	163 (78.0)	138 (69.0)	146 (71.6)	Reference			
CT	45 (21.5)	58 (29.0)	55 (27.0)	0.061	0.64 (0.40–1.02)	0.688	1.09 (0.71–1.7)
TT	1 (0.5)	4 (2.0)	3 (1.5)	0.282	0.30 (0.03–0.27)	0.603	1.04 (0.90–1.20)
Alleles							
C	371 (88.8)	334 (83.5)	347 (85.0)	Reference			
T	47 (11.2)	66 (16.5)	61 (15.0)	0.039^**∗**^	0.64 (0.42–0.98)	0.588	1.11 (0.76–1.63)
rs1799964 T>C							
Genotypes							
TT	127 (60.4)	137 (68.5)	142 (69.6)	Reference			
CT	71 (33.8)	56 (28.0)	58 (28.4)	0.176	1.36 (0.87–2.13)	0.863	1.04 (0.67–1.61)
CC	12 (5.7)	7 (3.5)	4 (2.0)	0.404	1.55 (0.56–4.31)	0.398	1.72 (0.49–6.07)
Alleles							
T	325 (77.3)	330 (84.4)	342 (83.8)	Reference			
C	95 (22.6)	70 (15.6)	66 (16.2)	0.134	1.32 (0.92–1.9)	0.543	1.12 (0.77–1.63)
rs1800629 G>A							
Genotypes							
GG	180 (85.7)	177 (88.5)	174 (85.3)	Reference			
GA	29 (13.8)	22 (11.0)	29 (14.2)	0.412	1.30 (0.70–2.40)	0.372	0.76 (0.42–1.38)
AA	1 (0.5)	1 (0.5)	1 (0.5)	-	-	0.904	0.84 (0.05–14.36)
Alleles							
G	389 (92.6)	376 (94.0)	377 (89.9)	Reference			
A	31 (7.4)	24 (6.0)	31 (10.1)	0.504	1.22 (0.68–2.19)	0.383	0.78 (0.45–1.36)
rs1800630 C>A							
Genotypes							
CC	135 (64.6)	145 (72.5)	151 (74.0)	Reference			
CA	65 (31.1)	50 (25.0)	49 (24.0)	0.132	1.42 (0.89–2.25)	0.693	1.10 (0.69–1.74)
AA	9 (4.3)	5 (2.5)	4 (2.0)	0.543	1.43 (0.45–4.56)	0.728	1.27 (0.33–4.84)
Alleles							
C	335 (80.1)	340 (85.0)	351 (86.0)	Reference			
A	83 (19.9)	60 (15.0)	57 (14.0)	0.130	1.34 (0.92–1.97)	0.608	1.12 (0.75–1.65)
rs361525 G>A							
Genotypes							
GG	196 (93.3)	189 (94.5)	194 (95.1)	Reference			
GA	14 (0.7)	11 (5.5)	9 (4.4)	0.911	1.05 (0.44–2.54)	0.592	1.28 (0.52–3.19)
AA	0 (0)	0 (0)	1 (0.5)	-	-	-	-
Alleles							
G	406 (96.7)	389 (97.3)	397 (97.3)	Reference			
A	14 (3.3)	11 (2.7)	11 (2.7)	0.912	1.05 (0.44–2.5)	0.935	1.04 (0.44–2.43)

SNPs, single nucleotide polymorphisms; CI, confidence interval; OR, odds ratio; PTB, pulmonary tuberculosis; LTBI, latent tuberculosis infection; HCS, healthy controls; # adjusted by age, sex status; ^*∗*^*P* < 0.05.

**Table 4:** Haplotypes of the* IL1B, IL6 and TNFα *genes and their distributions in the three groups.

Haplotype	PTB, *N* = 209 (%)	LTBI, *N* = 200 (%)	HCS, *N* = 204 (%)	PTB VS. LTBI *P* value	OR (95% CI)	LTBI VS. HCS *P* value	OR (95% CI)
*IL1B*							
CGA	23.06 (0.1)	33.20 (0.1)	35.71 (0.1)	0.120	0.65 (0.37–1.12)	0.750	0.92 (0.56–1.51)
CGG	230.90 (0.6)	192.64 (0.5)	198.30 (0.5)	0.039^*∗*^	1.34 (1.01–1.76)	0.690	0.94 (0.72–1.25)
GGA	158.98 (0.4)	169.95 (0.4)	161.77 (0.4)	0.200	0.83 (0.63–1.10)	0.560	1.09 (0.82–1.44)
*IL6*							
AA	221.06 (52.9)	200.16 (49.1)	3.91 (1.0)	0.701	1.06 (0.8–1.39)	0.390	1.13 (0.86–1.49)
AT	122.94 (29.4)	118.90 (29.7)	119.84 (29.4)	0.952	0.99 (0.73–1.34)	0.872	1.03 (0.76–1.39)
GT	64.06 (15.3)	66.10 (16.5)	82.16 (20.1)	0.656	0.92 (0.63–1.34)	0.197	0.79 (0.55–1.13)
*TNFα*							
CCGAG	81.97 (0.2)	58.98 (0.2)	55.94 (0.1)	0.070	1.40 (0.97–2.03)	0.660	1.09 (0.74–1.62)
CTACG	28.95 (0.1)	24.00 (0.1)	30.97 (0.1)	0.600	1.16 (0.66–2.03)	0.380	0.78 (0.45–1.35)
CTGCG	246.05 (0.6)	240.48 (0.6)	250.94 (0.6)	0.670	0.94 (0.71–1.24)	0.720	0.95 (0.71–1.27)
TTGCG	46.98 (0.1)	64.52 (0.2)	59.06 (0.2)	0.039^*∗*^	0.66 (0.44–0.98)	0.500	1.14 (0.78–1.68)
Other pooled^#^	29.00 (2.4)	24.10 (2.0)	18.32 (1.5)				

PTB, pulmonary tuberculosis; LTBI, latent tuberculosis infection; HCS, healthy controls; CI, confidence interval; OR, odds ratio; ^#^Total subjects which lowest frequency both in case and control less than 0.03; ^*∗*^*P* < 0.05.
